# Development and Evaluation of a Simplified CFD Model for a Swirl Diffuser with the Aid of 2D Particle Image Velocimetry

**DOI:** 10.1021/acsomega.5c12904

**Published:** 2026-02-19

**Authors:** Ondřej Šikula, Michal Krajčík, Pavel Zubík, Aliaksandra Mishuk, Müslüm Arici

**Affiliations:** † Faculty of Civil Engineering, 48274 Brno University of Technology, Brno 601 90, Czech Republic; ‡ Institute of Construction and Architecture, Slovak Academy of Sciences, Bratislava 845 03, Slovakia; § Faculty of Civil Engineering, Slovak University of Technology in Bratislava, Bratislava 812 43, Slovakia; ∥ Engineering Faculty, Kocaeli University, Kocaeli 41001, Turkey

## Abstract

In numerical modeling of swirl diffusers, simplifying the diffuser geometry can substantially reduce simulation costs. This study describes the development and performance evaluation of simplified computational fluid dynamics (CFD) models of a swirl diffuser by using 2D particle image velocimetry (PIV). Two simplified models were created by replacing the diffuser with simple supply openings, where momentums were prescribed. The first model defined momentums solely based on geometric rules. In the second, it was demonstrated how the accuracy of the first, geometry-based model, can be improved by adjusting the momentums using results from a detailed CFD model. The effect of using the Reynolds stress model or the SST *k-ω* turbulence model on the accuracy of the CFD model and computation time was investigated. The simplified models were benchmarked against both the detailed CFD model and the PIV measurements. The corrected simplified geometry combined with the SST *k-ω* turbulence model achieved a favorable balance between reliability and computational efficiency. Although the two-dimensional nature of the PIV measurements presented challenges for three-dimensional CFD validation, they proved valuable for developing and assessing the simplified models.

## Graphical abstract

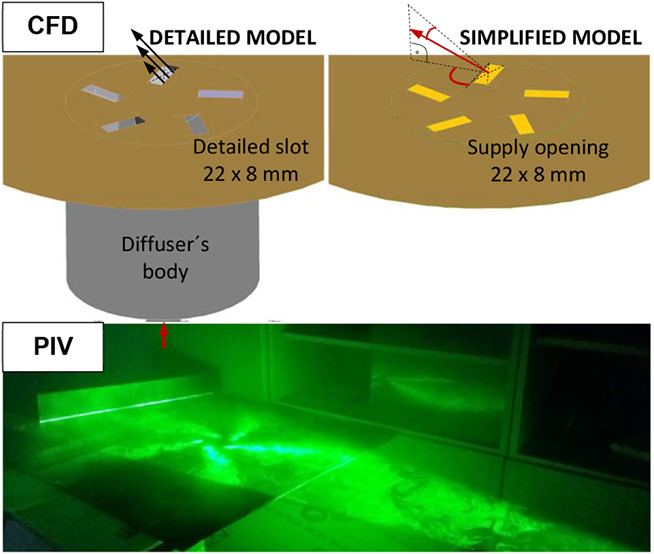

## 1 Introduction

Swirl diffusers are among the most popular and frequently used air terminal devices because they can meet high standards of indoor air quality and thermal comfort. This is achieved by an intense swirling motion that diminishes the influence of gravity, promotes the mixing of room air, and creates a homogeneous thermal environment.^1,2^

Direct measurement of airflow generated by swirl diffusers is time-consuming and often difficult. While product catalogs provide basic design parameters, atypical spaces or those with special indoor-environment requirements demand more detailed information. Computational fluid dynamics (CFD) offers an affordable means of visualizing airflow in such cases.^3,4^ However, preparing and running a detailed CFD model can be computationally expensive, particularly because supply openings typically have complex geometries and require dense computational grids.^5^ Consequently, complex swirl-diffuser models are often replaced with simplified geometries and boundary conditions.

Two common approaches for the simplified representation of supply openings are the box method and the momentum method. The box method applies boundary conditions on the surface of a control volume placed some distance from the diffuser. Although this approach can save computation time, it requires experimental data and more effort to define the boundary conditions.^6,7^ The momentum method, in contrast, prescribes radial, tangential, and axial momentum components at one or more simplified openings that represent the diffuser slots. This method is easier to implement and is particularly suitable for diffusers discharging combined jets, such as swirl diffusers.^7^ It is the most frequently used method in CFD modeling of swirl diffusers, as shown in Table 1 which summarizes studies on CFD modeling of swirl diffusers and compares them with the present study.

**Table 1:** Research Studies That Involve CFD Modeling of Swirl Diffusers^b^

Study	Turbulence model	Computational domain	Diffuser location	Evaluated parameters	Measured, validated	Supply temperature	Simplified boundary conditions at supply opening
Hu^10^	*k-ε*	Diffuser	Ceiling	*v*_air_, *Tu*	*v*_air_	Isothermal	Detailed model of swirling vanes. Airflow in supply duct included
Xu and Niu^11^	*k-ε*	Room	Floor	*v*_air_, θ_air_	-	Cool	Detailed model of swirling vanes. Airflow in supply duct included
Wang and Pepper^12^	RNG *k-ε*	Room	Floor	*v*_air_, θ_air_	-	Cool	Simplified diffuser geometry. Details not reported
Zhou and Haghighat^13^	RNG *k-ε*	Room	Floor	*v*_air_, θ_air_, *c*_*CO2*_	*v*_air_, θ_air_, *c*_*CO2*_	Cool	Momentum modeling at air supply: diffuser divided into 6 sectors, momentum origin in each sector
Zhang et al.^9^	RNG *k-ε*	Room	Floor	*v*_air_, θ_air_, *c*_*SF6*_	*v*_air_, θ_air_, *c*_*SF6*_	Warm	Simplified diffuser geometry. Axial and tangential velocity modeled. Actual velocities randomly assigned on a ratio of CFD cells, equal to *A*_eff_
Rusly and Piechowski^14^	*k-ε*	Room	Ceiling	*v*_air_, AoA	-	Cool	Momentum modeling at air supply: diffuser divided into 16 parts, momentum origin in each part
Sajadi et al.^15^	*k-ε*	Room	Ceiling	*v*_air_, ET, ER, jet decay coeff.	-	Isothermal	Modeled 12 slots including their geometry. Details not specified
Aziz et al.^16^	*k-ε* (*k-ω*, RNG *k-ε*)^a^	Room	Ceiling	*v*_air_, θ_air_, RH, *ε*_*v*_, AoA, EDT	θ_air_	Cool	Not specified
Koskela and Maula^8^	*k-ε*, SST	Room	Ceiling	*v*_air_	*v*_air_	Cool, isothermal	Momentum modeling at air supply: momentum origin in each of 8 or 9 simplified openings
Tavakoli and Hosseini^17^	LES	Room	Ceiling	*v*_air_, AoA	-	Not specified	Momentum modeling at air supply: annular inlet, axial and tangential momentum defined by swirl angle
Liu et al.^18^	LES	Diffuser	Floor	*v*_air_, θ_air_, AoA, TKE, momentum balance	-	Not specified	Momentum modeling at air supply: momentum origin in center of diffuser
Yau et al.^19^	*k-ε* (RNG *k-ε*, realizable *k-ε*)^a^	Diffuser	Floor	*v*_air_, θ_air_	*v*_air_	Cool	Not specified
Abdolzadeh et al.^20^	v^2^-f	Room	Floor	*v*_air_, θ_air_	*Particle concentration*	Cool	Momentum modeling at air supply: momentum origin in center of diffuser using three velocity components
Borowski et al.^21^	*k-ε*	Diffuser	Horizontal	*v*_air_	*v*_air_	Isothermal	Detailed model of swirling vanes. Airflow in supply duct included
Taheri et al.^22^	*k-ε*	Room	Floor	*v*_air_, θ_air,_ *particle concentration and deposition*	*v*_air_, θ_air_, *particle concentration*	Cool	Diffuser divided into nine small squares. Center square defined as a wall. Air velocity on eight other squares were assigned different directions and angles.
Bennia et al.^23^	LES	Diffuser	Ceiling	*v*_air_	*v*_air_	Isothermal	Detailed model of swirling vanes. Airflow in supply duct not included
Present study	RSM, SST *k-ω*	Diffuser	Ceiling, floor	*v*_air_, *Tu*	*v*_air_, *Tu*	Isothermal	Momentum modeling at air supply: 5 slots, each represented by axial, radial, and tangential velocity

a The turbulence models in parentheses were also investigated, but only to a limited extent.

b **Abbreviations:** AoA: age of air; EDT: effective draft temperature; ER: entrainment ratio, ET: evacuation time; RH: relative air humidity. **Symbols:** *A*_*eff*_: effective area of the air supply diffuser (m^2^); *c*_*CO2*_: concentration of CO_2_ (ppm); *c*_*SF6*_ - concentration of SF_6_ (ppm); *Tu:* turbulence intensity (%); *v*_air_: air velocity magnitude (m.s^–1^); *ε*_*v*_: ventilation effectiveness (-); θ_air_: room air temperature (°C).

For reliable results, the momentum vectors in a simplified model must be adjusted so that the computed airflow field reproduces reality. Some guidance for such adjustment has been provided by, for example, Koskela and Maula,^8^ and Zhang et al.^9^ Koskela and Maula^8^ applied the momentum method to develop a simplified model of a swirl diffuser adjustable between a radial swirl jet for cooling and a downward swirl jet for heating. Zhang et al.^9^ proposed a novel procedure for simulating floor-level swirl diffusers, randomly assigning actual velocities to a proportion of CFD cells equivalent to the diffuser’s effective area and prescribing axial and tangential components from experimental data. Nevertheless, the existing scientific articles summarized in Table 1 offer limited detail on geometry creation, boundary-condition adjustment, or the choice of an appropriate turbulence mode.

Experimental validation is essential to building confidence in CFD simulations. In half of the 16 studies listed in Table 1, air velocity measurements were used for validation, almost always with traditional point-wise anemometry. While useful, this technique makes accurate measurement of turbulent flows challenging.^24^ Particle image velocimetry (PIV) provides an attractive alternative because it can capture detailed velocity fields in mechanically ventilated spaces without disturbing the airflow.^25,26^ However, PIV is rarely applied to full-scale ventilation systems and is more feasible for small-scale models if scaling effects are addressed.^24^ Applications of PIV to validate CFD simulations of building ventilation are limited and have focused mainly on natural ventilation.^27–30^

Based on the review of scientific literature, three key research gaps emerge in CFD modeling of swirl diffusers aided by PIV:

- Existing studies give little guidance on constructing reliable simplified CFD models of swirl diffusers.
- Reports of PIV measurements for swirl diffusers remain scarce.
- The use of PIV for validating CFD models of any type of air-supply diffuser has not been thoroughly documented.

This study addresses these gaps by presenting a systematic procedure to develop a reliable simplified CFD model of a swirl diffuser, demonstrating how such a model can save significant time in modeling, meshing, and computation. The work also evaluates the use of PIV for assessing the CFD performance. The momentum method was selected for describing the diffuser slots because it conserves mass and momentum while avoiding the need for detailed boundary-condition data required by the box method. We describe how to correct momentum components and boundary conditions to achieve a simplified CFD model. As an essential first step toward predicting the airflow in an entire room, we focus on the near-diffuser region.

Section 2 outlines the procedure for developing simplified models of the swirl diffuser. Section 3 details the PIV measurement methodology used to assess the accuracy of both the detailed and simplified CFD models. Section 4 describes the creation and parameter settings of the CFD models. Section 5 defines the indices used to evaluate the CFD model accuracy with the aid of PIV, and Section 6 presents the evaluation results. Sections 7 and 8 discuss the findings, draw conclusions, and offer recommendations for the development and evaluation of CFD models of swirl diffusers.

## 2 Procedure for Developing Simplified CFD Models of the Swirl Diffuser

Figure 1 summarizes the sequence of steps used to develop and evaluate simplified CFD models. The process was as follows: PIV measurements were done (1), and a detailed CFD model (DM) was created (2). The DM results were compared with the PIV data (3), and a good agreement was found, as shown in Section 6 and discussed in Section 7, thereby validating the DM (4). The turbulence intensity at the slots was calculated for use in the simplified models (5). We developed a geometry-based simplified model (SMG), as explained in Section 4.5 (6). The SMG results were then compared to those from the DM and, by extension, to the PIV (7). Upon comparison, differences between SMG and DM were identified, and the agreement was deemed insufficient (8). The momentum angles in the SMG were therefore adjusted (9), resulting in a corrected simplified model (SMC), as detailed in Section 4.6 (10). When the CFD models were finalized, their accuracy was evaluated using the PIV measurements and, for the simplified models, also by comparison with the DM results by applying the indicators defined in Section 5.

**Figure 1:**
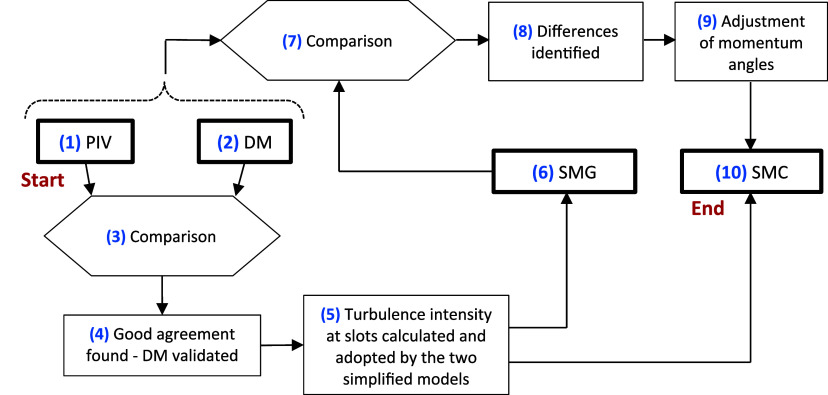
Procedure for developing and evaluating simplified CFD models of a swirl diffuser.

## 3 Methodology of PIV Measurements

This section describes the procedure used for the PIV measurements. The PIV results were subsequently employed to evaluate the performance of the CFD models, as presented in Section 6.

### 3.1 Experimental Model of the Swirl Diffuser

The face of the swirl diffuser had angled slots arranged in a circle and adjustable radial pattern (Figure 2). The diffuser diameter was 100 mm, making it particularly suitable for localized ventilation and low air-supply flow rates. In this study, the applicability of PIV for evaluating CFD models was demonstrated using a slot angle of 30° and an air-supply flow rate of 3.11 l.s^–1^.

**Figure 2:**
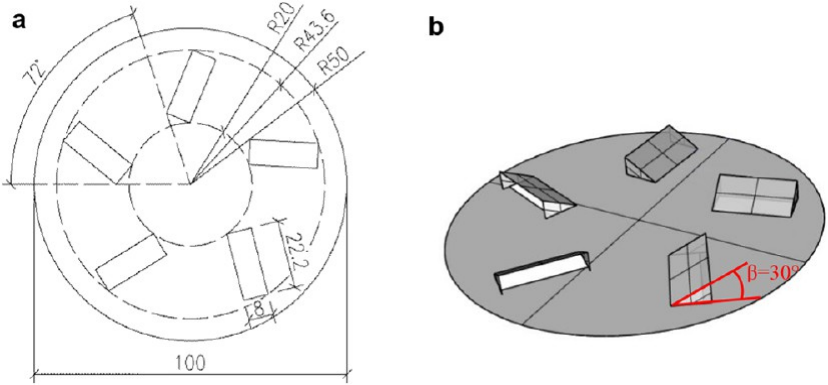
Face of the swirl diffuser: a) dimensions and b) slots.

### 3.2 Experimental Setup and Measurement Procedure

The swirl diffuser was mounted in a wooden leveling panel painted black to enhance the visibility of the seeding particles from the glycerin fog used to visualize the airflow (Figure 3a). A propeller anemometer embedded into the supply duct measured the air supply flow rate. The air supply was maintained under isothermal conditions, similar to the setups described by Hu,^10^ Sajadi et al.,^15^ and Koskela and Maula,^8^ with an average temperature of 23.5 °C. The effect of air supply temperature was considered negligible because nearby the diffuser, the effect of gravitational forces is small compared with the momentum forces of forced convection that dominate the airflow (Gr/Re^2^ < 1). Furthermore, even under nonisothermal conditions, the high mixing rate in the initial flow would rapidly diminish temperature differences, thereby further reducing buoyancy effects.^1^

**Figure 3:**
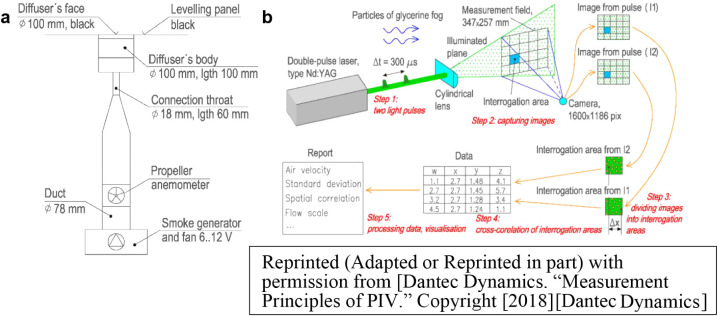
a) Experimental model of the swirl diffuser and b) procedure of PIV measurements^31^

The procedure for the PIV measurements is illustrated in Figure 3b. Initially, a controlled amount of glycerin fog was uniformly distributed throughout the measurement volume to avoid excessively high particle concentrations near the diffuser slot and low concentrations farther away. The remaining fog was supplied through the air diffuser at a constant rate. The average droplet size in the fog was approximately 1 μm. A dual-pulse laser emitted two consecutive light pulses to create an illuminated plane parallel to the diffuser face. The beam passed through a cylindrical lens and illuminated the fog particles. The measurement equipment consisted of:

- Nd:YAG double-pulse laser (Gemini PIV, New Wave Research) with adjustable repetition rate from 0 to 15 Hz, wavelength 532 nm, and maximum energy of 120 mJ.
- FlowSense 2 M (Dantec) with a resolution of 1600 × 1200 pixels and a Micro-Nikkor 60 mm lens.
- System Hub (Dantec) with FlowManager v4.71 software for synchronized operation of all components.
- Hurricane 1200 (Chauvet) with output power regulation to maintain a stable fog concentration.

A photograph of the airflow field visualized by the glycerin fog during a laser pulse is shown in Figure 4a. The camera captured the instantaneous positions of the fog particles during each light pulse. The images were analyzed using adaptive cross-correlation^32^ in three steps: from an initial interrogation-window size of 128 × 128 pixels, progressively refined to 32 × 32 pixels. In each step, interrogation areas overlapped by 25% and were cross-correlated at the pixel level. The resulting correlation peak identified the displacement (Δ) of individual seeding particles. This procedure was repeated 150 times at a frequency of 15 Hz. The final output was a velocity field represented as a vector map containing 66 × 44 vectors (Figure 4b).

**Figure 4:**
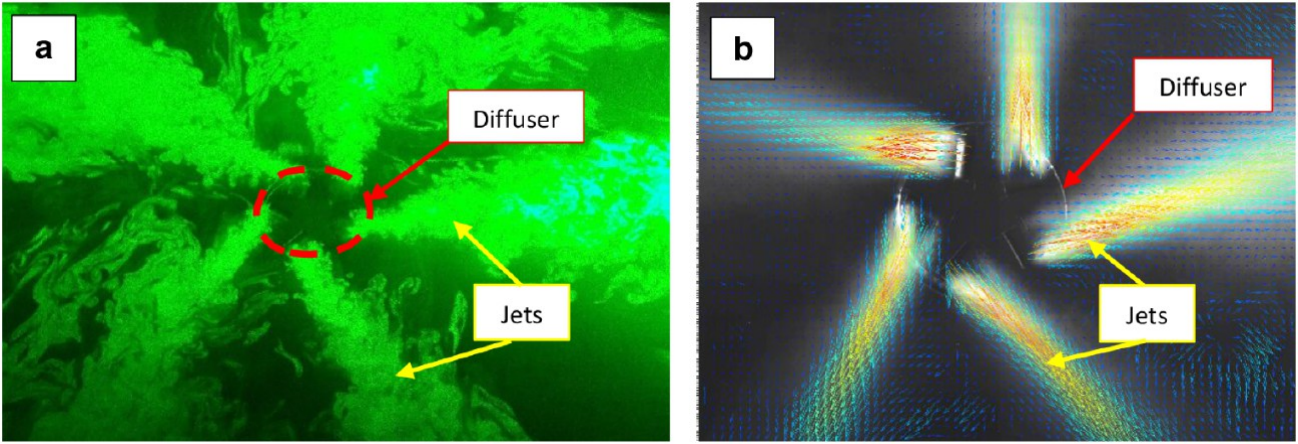
a) Photograph of the airflow field visualized by glycerin fog during a laser pulse. Although the photo was taken at an angle, the illuminated plane used to capture particle movement was parallel to the diffuser face. (b) Velocity vector field 10 mm above the diffuser obtained from PIV measurements.

All PIV measurements were conducted at three horizontal planes located 10, 20, and 30 mm above the diffuser (Figure 5). The experiments took place in a room with stable air temperature, where air exchange with the surrounding environment was minimized and, therefore, considered negligible. Windows were completely covered with aluminum foil, and all lights were switched off. The diffuser was isolated from external influences that could disturb the airflow field. All walls and potential obstacles were positioned at least 100 times the diffuser’s diameter away, ensuring that any secondary airflow was induced primarily in the axial direction and remained unobstructed.

**Figure 5:**
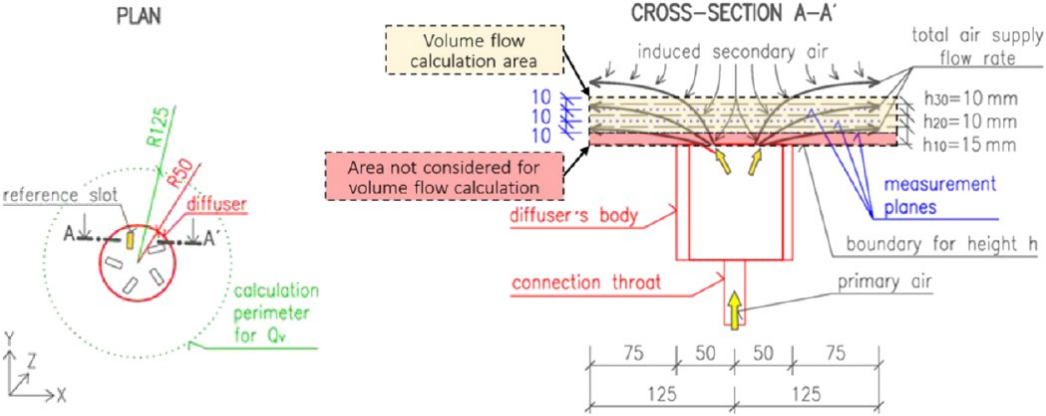
Definition of measurement planes and heights.

### 3.3 Determination of the Total Supply Airflow Rate (Q_v_)

The PIV measurements were used to determine the total supply airflow rate (*Q*_v_), defined as a sum of the primary air (3.11 l.s^–1^) and the induced secondary air (Figure 5). The total airflow rate was calculated from the PIV data for a radius of 125 mm (R125).

Figure 6 presents the three two-dimensional air-velocity fields (excluding the axial velocity component) obtained from PIV measurements and used to calculate the total supply airflow rate at measurement planes located 10, 20, and 30 mm above the diffuser face. Minor asymmetries in the jets are visible despite careful experimental preparation and execution. For the flow-rate calculation, the PIV-derived velocity field was divided into a series of cells. The cells intersecting the jets along the perimeter of each measurement plane were used to compute the total supply airflow rate.

**Figure 6:**
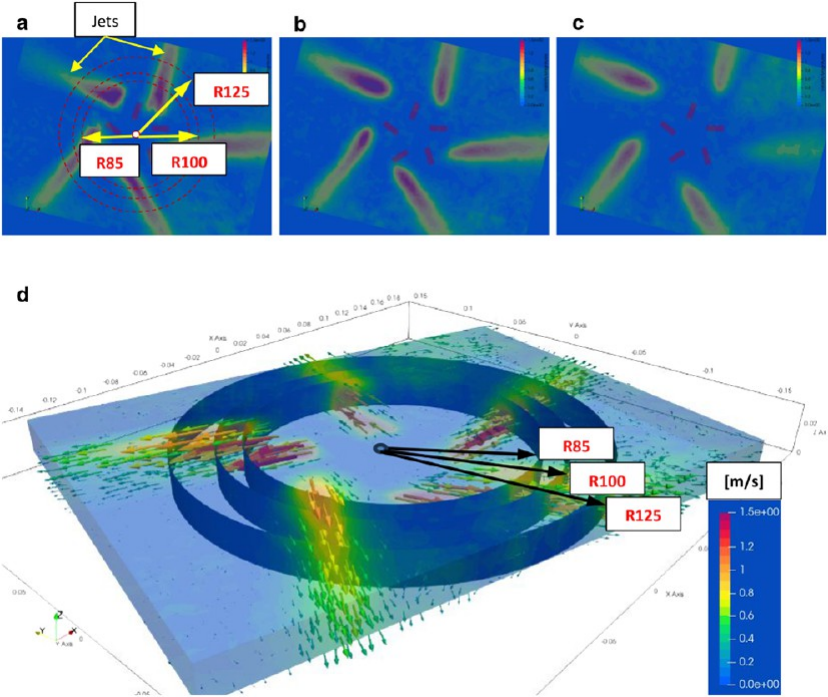
Air velocity fields used for calculation of total supply airflow rate: a) 10 mm, b) 20 mm, and c) 30 mm above the diffuser face, d) 3D velocity vectors and definition of measurement radii.

Each calculation cell was defined by its horizontal dimensions and the corresponding height *h*_*10*_, *h*_*20*_ or *h*_*30*_. The airflow rate was determined using only the horizontal (x, y) velocity components because, owing to the two-dimensional nature of the PIV measurements, detailed information about the vertical (z) velocity component was unavailable. The total air-supply flow rate was calculated as:

1 $$Q_{V,10}=\sum \left(v_{y,10}\times \Delta x_{i,10}+v_{x,10}\times \Delta y_{i,10}\right)\times 0.01$$

2 $$Q_{V,20}=\sum \left(v_{y,20}\times \Delta x_{i,20}+v_{x,20}\times \Delta y_{i,20}\right)\times 0.01$$

3 $$Q_{V,30}=\sum \left(v_{y,30}\times \Delta x_{i,30}+v_{x,30}\times \Delta y_{i,30}\right)\times 0.01$$

4 $$Q_{V}=Q_{V,10}+Q_{V,20}+Q_{V,30}$$

where *Q*_*v,10*_, *Q*_*v,20*_, and *Q*_*v,30*_ are the air volume flow rates through the regions defined by *h*_*10*_, *h*_*20*_ and *h*_*30*_, respectively, Δ*x*_*i*,*10*_, Δ*y*_*i,10*_, Δ*x*_*i*,*20*_, Δ*y*_*i,20*_, and Δ*x*_*i*,*30*_, Δ*y*_*i*,*30*_ denote the dimensions of the cell *i* in the *x* or *y* direction at 10, 20, and 30 mm above the diffuser face, respectively, *v*_*y*,10_, *v*_*x,*10_, *v*_*y,*20_, *v*_*x,*20_, and *v*_*y,*30_, *v*_*x,*30_ represent the scalar air velocities at the cell *i* in the *x* or *y* direction at 15, 25, and 35 mm above the diffuser’s face, respectively. The calculations were performed in the software Paraview 5.10. It should be noted that the values of *Q*_*v,10*_, *Q*_*v,20*_, and *Q*_*v,30*_ are underestimated due to the dimensional limitations of the PIV measurement.

## 4 Numerical Models for CFD Simulations

The numerical models were solved by ANSYS Fluent software, release 18.2. The diffuser slot angle and supply airflow rate were 30° and 3.11 l·s^–1^, respectively, matching the PIV measurements.

### 4.1 Governing Equations and Cases Investigated

The fluid flow from the swirl diffuser is generally described by the advection-diffusion equation, expressed as the continuity equation together with the three Navier–Stokes momentum equations needed to describe the isothermal flow. To account for turbulence, each quantity in the advection–diffusion equation was decomposed into time-averaged and fluctuating components, yielding the Reynolds-averaged Navier–Stokes (RANS) equations, which were solved to compute the air-velocity field.^5,33^ The equations were discretized by using second-order schemes and a double-precision solver. Iterations continued until the residuals satisfied a convergence criterion of 10^–4^ and the maximum outlet velocity stabilized. To examine the influence of turbulence modeling on CFD performance, the Reynolds shear stress tensor was represented using two different turbulence models:

(1) the precise but computationally demanding Reynolds stress model (RSM), specifically the stress-BSL formulation, which directly computes each component of the Reynolds stress tensor,^34^
(2) the supposedly less accurate but less demanding Shear Stress Transport *k-ω* (SST *k-ω*) two-equation turbulence model is based on the Boussinesq eddy viscosity assumption.^35,36^

Referring to Table 1, the standard *k-ε* is the turbulence model that has been used most frequently in CFD modeling of swirl diffusers. In this study, SST *k-ω* was selected as a representative two-equation model because it combines the strengths of the Standard *k-ω* model near walls with those of the Standard *k-ε* model in the free stream. A known limitation of SST *k-ω* is its assumption of turbulence isotropy, i.e., equal treatment of the fluctuating velocity components *ú*, *v́*, and *ẃ*, which may be restrictive for swirling flows.

Unlike the simplified models, the RSM is the most detailed turbulence model available in ANSYS Fluent. It avoids the Boussinesq eddy-viscosity assumption and directly solves transport equations for each Reynolds stress component, allowing for turbulence anisotropy.^37^ Although rarely applied in previous swirl-diffuser studies (see Table 1), RSM was investigated here to assess whether its higher computational cost yields improved accuracy and is therefore practical for the CFD simulation of swirl diffusers.

Two pressure-velocity coupling algorithms provided in ANSYS Fluent were also compared for their effect on computational stability and time: the Semi-Implicit Method for Pressure-Linked Equations (SIMPLE), which uses a segregated approach,^38^ whereas the Coupled algorithm solves the continuity and momentum equations simultaneously.

Figure 7 illustrates the six combinations of geometry, CFD model, and turbulence model. The detailed model (DM) represents the CFD simulation with a full geometric fidelity. The SMG is a geometry-based simplified model (Section 4.5), while the SMC is a simplified model corrected using DM results and PIV measurements for improved accuracy (Section 4.6). Each CFD model was evaluated using both turbulence models, SST *k-ω* and RSM, and the two pressure-velocity coupling algorithms were compared in terms of computation time.

**Figure 7:**
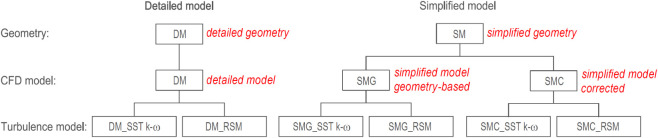
Cases investigated.

### 4.2 Geometry

The geometries of the three CFD models are shown in Figure 8. The detailed CFD model replicates the exact shape and dimensions of the physical diffuser. In the simplified CFD models, the connection throat, diffuser body, and slots were replaced by five rectangular supply openings with dimensions of 22 mm x 8 mm. In the geometry-based simplified model (SMG), the momentum angle was set according to geometric rules (Section 4.5). The corrected simplified model (SMC) was derived by adjusting the horizontal and vertical momentum angles of the SMG based on the detailed CFD model (Section 4.6). This correction aimed to improve the predictive accuracy of the simplified model.

**Figure 8:**
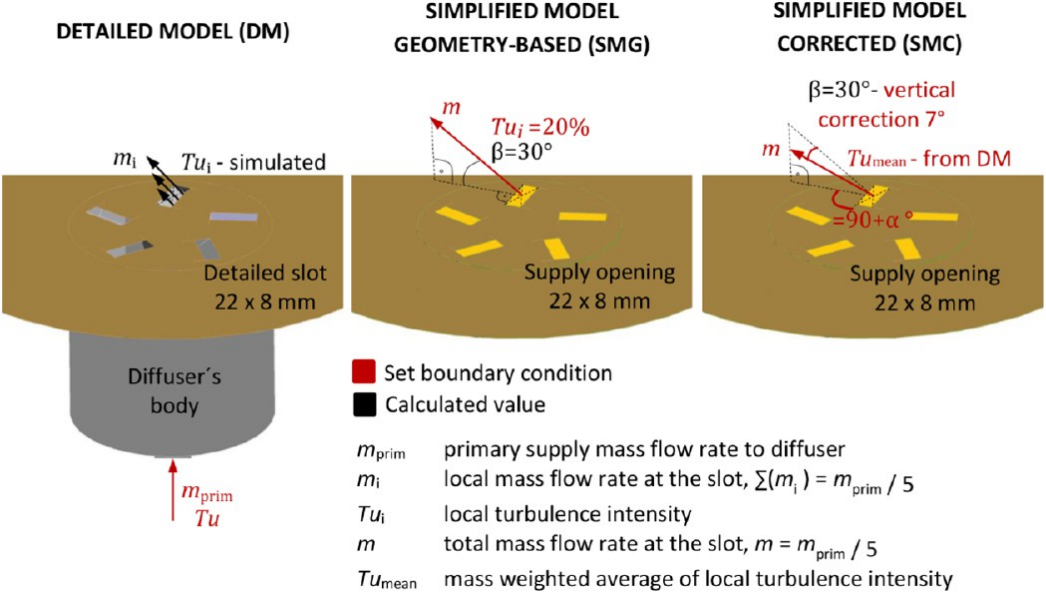
Geometry of the three CFD models of the swirl diffuser.

### 4.3 Computational Mesh

An unstructured tetrahedral mesh generated in the ANSYS Meshing was used to capture the complex geometry of the diffuser. The simulation domain was divided into four zones, indicated in Figures 9 and 10 by colored circles with radii R85, R100, and R125. These radii were required to calculate the total supply airflow rate in the CFD simulations, as explained in Section 3.3.

**Figure 9:**
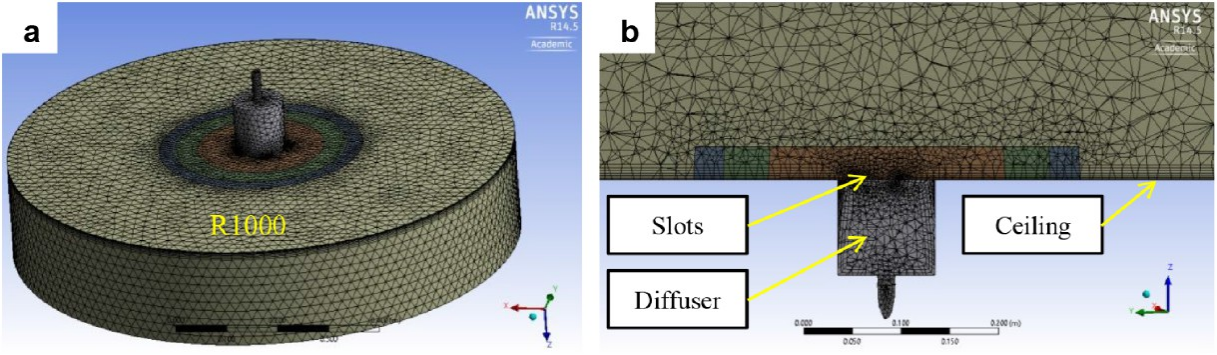
Computational mesh of detailed model: a) overall view, b) detail.

**Figure 10:**
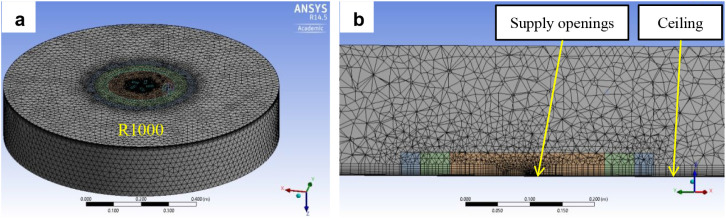
Computational mesh of simplified models: a) overall view, b) detail.

Prismatic boundary layers were applied to the ceiling, outer surfaces of the diffuser, and the slots. The maximum grid-cell size was limited to maintain a very fine mesh. In both the detailed model (Figure 9) and the simplified model (Figure 10), the mesh was progressively refined toward the diffuser slots. In particular, for the simplified models, fine meshing at the supply openings was critical to ensuring reliable results (Figure 11). In Figure 11, the upper pictures show the original computational mesh at supply openings from ANSYS Meshing. In Fluent, the tetrahedral mesh was converted to a polyhedral mesh, as shown in the lower pictures.

**Figure 11:**
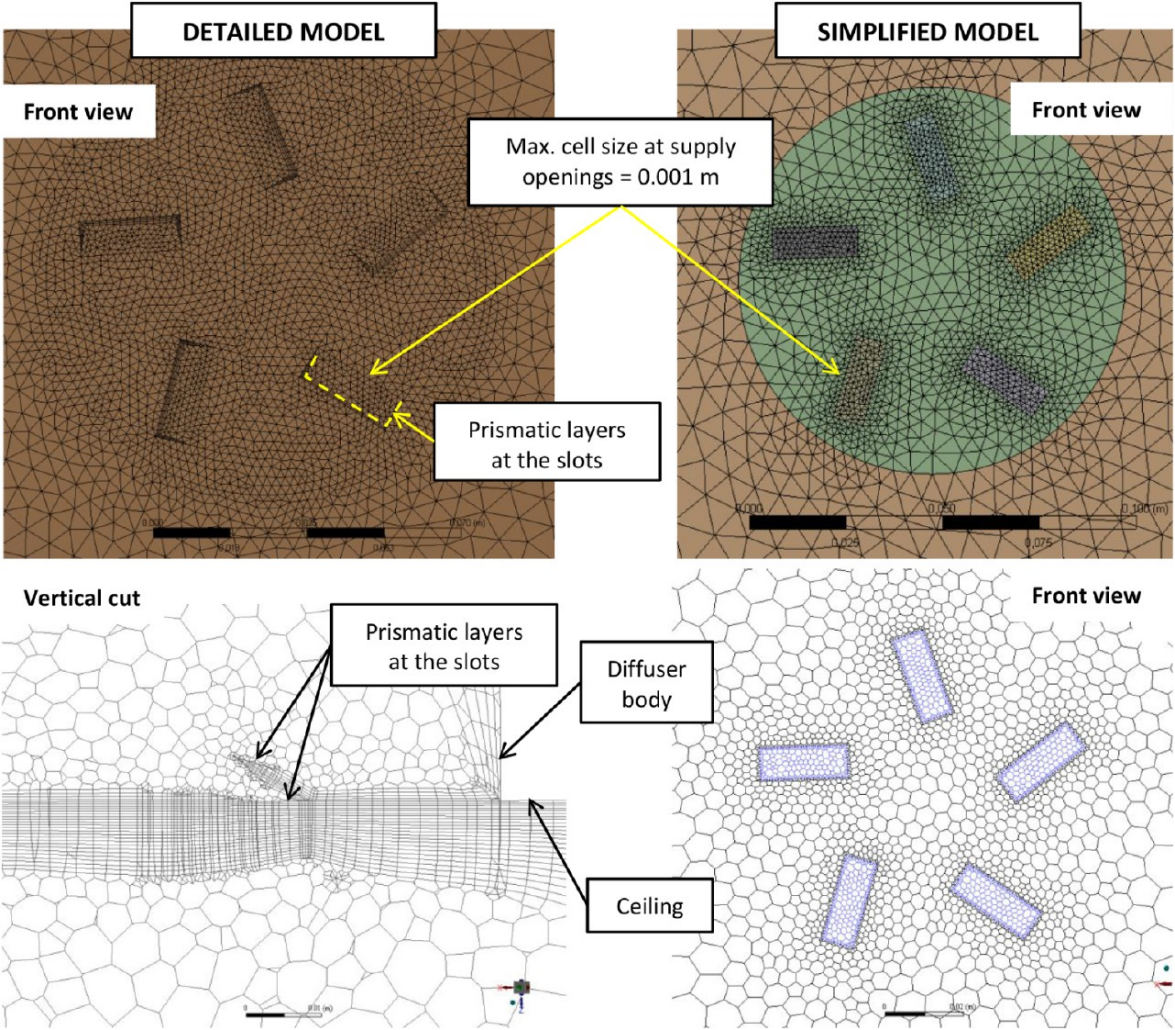
Computational mesh at supply openings.

Grid independence was verified through successive mesh refinements. Initially, the grid was refined by doubling the number of cells at the inlet. Subsequently, the grid was further refined by doubling the number of cells over the rest of the surface of the leveling panel. In total, three levels of the computing mesh were compared. Changes in the average values of three performance indicators were observed: turbulence intensity (%), cell Reynolds number (-), and vorticity (rad.s^–1^). The differences between the finer and finest meshes were smaller than those between the original and finer meshes and ranged from 0.8 to 1.7% for all indicators. It was therefore concluded that further refinement would have only a minor effect on these performance indicators.

Table 2 presents the computational mesh characteristics for both the detailed and simplified models expressed using several quality metrics. Although simplifying the diffuser geometry resulted in only a modest reduction in the total number of computational cells, it provided significant advantages. Most notably, it greatly reduced the time required to construct both the complex diffuser geometry and the computational grid. Moreover, the quality indicators in Table 2 demonstrate that, for a comparable number of cells, the simplified geometry allowed the creation of a mesh higher in quality than the detailed geometry.

**Table 2:** Characteristics of the Computational Mesh

Detail of the CFD model		Detailed (DM)	Simplified (SMG, SMC)
No. of computational cells		274 919	266 022
Orthogonal quality; range: 0–1, best quality = 1	Minimum	0.072	0.272
	Average	0.918	0.877
Aspect ratio; recommended maximum 20–60	Maximum	26.99	32.267
	Average	4.12	3.484
Skewness; range: 0–1	Maximum	0.858	0.796
	Average	0.247	0.223

### 4.4 Boundary Conditions

Figure 12 illustrates the assignment of boundary conditions in the CFD models. In the detailed model (a), the mass-flow inlet was modeled explicitly, whereas in the simplified models (b), it was applied directly to the five openings representing the diffuser slots. The specific boundary conditions are summarized in Table 3. Apart from these differences, all other boundary conditions were identical. For the simplified models, the radial, tangential, and axial momentum components were specified at the inlet openings.

**Figure 12:**
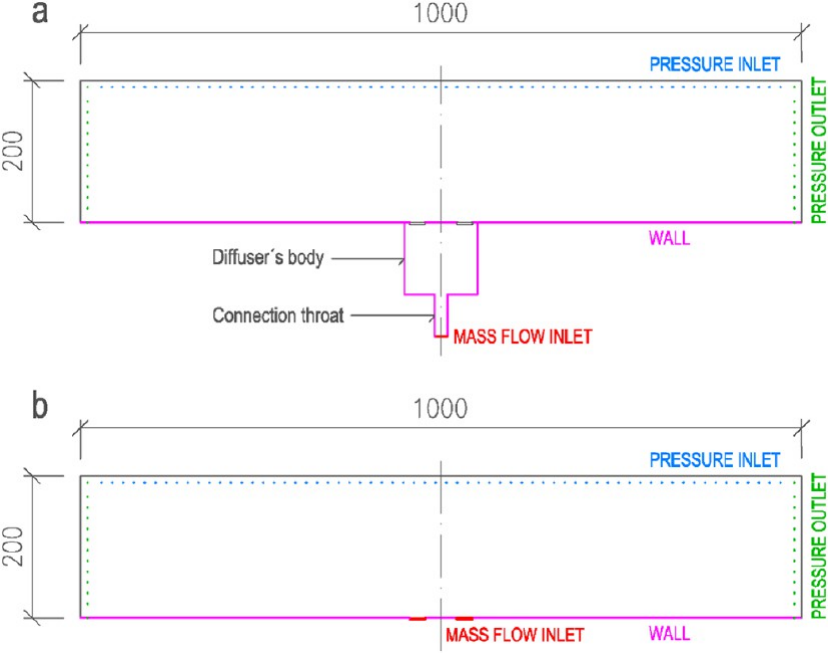
Assignment of boundary conditions: a) detailed model and b) simplified models.

**Table 3:** Overview of the Boundary Conditions

Item	Boundary condition	Detailed (DM)	Simplified**—**geometry-based (SMG)	Simplified**—**corrected (SMC)
Gravitational forces	*g* = −9.81 m.s^–2^	no		
Fluid	Density (kg.m^–3^)	1.18		
	Viscosity (kg.m^–1^.s^–1^)	1.79.10^–5^		
Outlet	**Vertical— pressure outlet**			
	Condition type	Pressure outlet		
	Turbulence intensity (%)	5		
	Turbulent length scale (m)	0.01		
	**Horizontal—pressure inlet**			
	Condition type	Pressure outlet		
	Turbulence intensity (%)	5		
	Turbulent length scale (m)	0.01		
Inlet (supply)	Mass-flow inlet (kg.s^–1^)	0.003665		
	Turbulence intensity (%)	5	20	20
	Hydraulic diameter (m)	0.08	0.01173	0.01173
	Ratio of radial momentum (-)	-	0.310356	0.474095
	Ratio of tangential momentum (-)	-	0.808504	0.789027
	Ratio of axial momentum (-)	-	0.5	0.390731

In the detailed model, the mass flow rate and turbulence intensity were specified at the inlet of the connection throat. A low turbulence intensity of 5% was applied because the throat length was sufficient to establish a realistic air-velocity and turbulence field at the diffuser inlet. The complex geometry and more precise boundary condition specification in the detailed model produced an uneven distribution of airflow characteristics at the reference slot (Figure 13). This distribution is more realistic than that in the simplified models, where the airflow characteristics are represented by averaged values.

**Figure 13:**
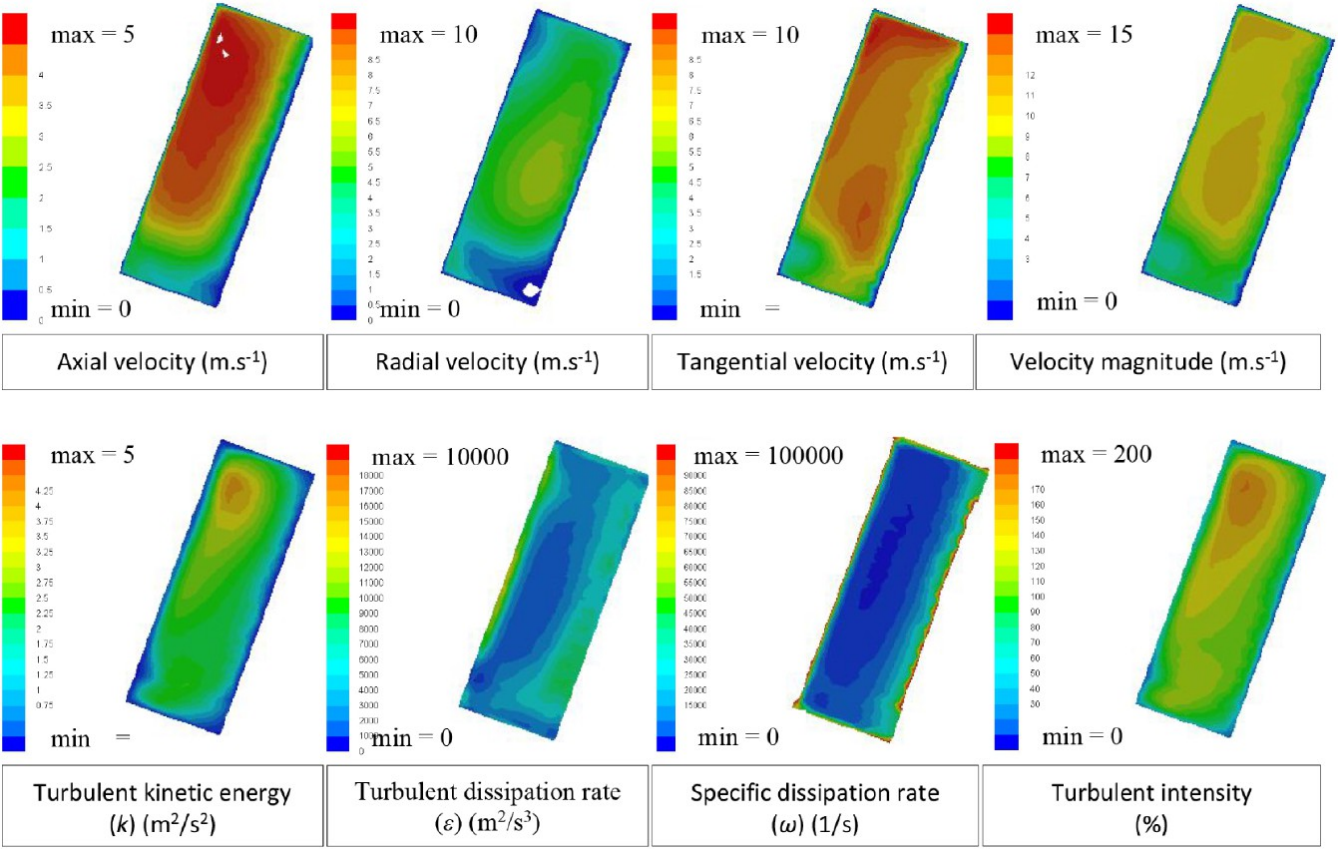
Air velocity and turbulence characteristics of the detailed CFD model at the reference slot.

In the simplified models, the diffuser body was not included. The turbulence intensity was specified at the supply openings, where the flow field is more complex and turbulent than that in the connection throat. Consequently, a higher turbulence intensity of 20% was assigned to the simplified models, compared with 5% for the detailed model. The turbulence intensity (*Tu*) used at the slots in the simplified models is based on the average velocity v̅ = 9.15 m/s and the average turbulent kinetic energy at the slots *k* = 4.92 m^2^/s^2^ of the detailed model according to the relationship *Tu* = (2*k*/3)^0.5^/v̅ ≈ 20%. Finally, the hydraulic diameter was determined as:

5 $$d_{h}=4\frac{S}{P}=4\frac{\left(\frac{22}{1000}\times \frac{8}{1000}\right)}{2\frac{22}{1000}+2\frac{8}{1000}}=0.01173$$

where *d*_*h*_ is the hydraulic diameter (m), *S* is the area of the opening (m^2^), and *P* is the perimeter of the opening (m).

### 4.5 Creation of Geometry-Based CFD Model (SMG)

To develop a reliable simplified model, it is essential to accurately define the airflow direction at the supply openings. In the SMG, the axial, radial, and tangential momentum components were adjusted strictly according to geometric rules, as illustrated in Figure 14. Assuming the magnitude of the resultant momentum mmm equals 1, the ratios of the radial (*m*_r_), tangential (*m*_t_), and axial (*m*_a_) momentum components were determined as *m*_r_ = 0.310356, *m*_t_ = 0.808504, and *m*_a_ = 0.5, using the following equations:

6 $$m_{h}=m\cdot \cos\;\beta$$

7 $$m_{a}=m\cdot \sin\;\beta$$

8 $$m_{t}=m_{h}\cdot \sin\;\alpha =m\cdot \cos\;\beta \cdot \sin\;\alpha$$

9 $$m_{r}=m_{h}\cdot \cos\;\alpha =m\cdot \cos\;\beta \cdot \cos\;\alpha$$

**Figure 14:**
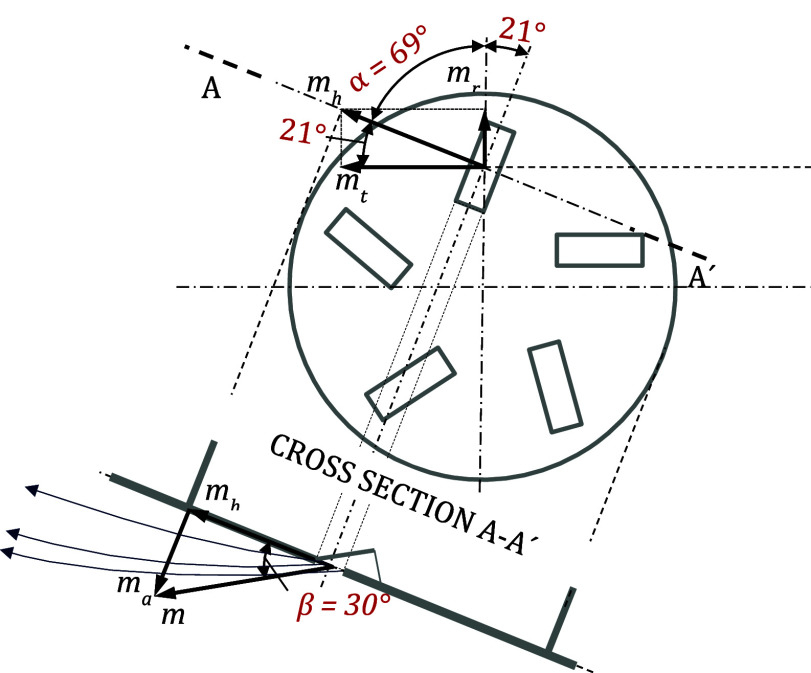
Air velocity momentums determined by geometric rules: *m*_r_: radial momentum, *m*_t_: tangential momentum, *m*_a_: axial momentum, *m*_h_: horizontal component of the resultant momentum, and *m*: resultant air velocity momentum.

### 4.6 Correction of SMG by Detailed CFD Model to Obtain SMC

The geometry of SMC is identical with the geometry of SMG. However, in SMC, the horizontal and vertical components of the resulting momentum were corrected based on the results of CFD simulations for the detailed model. The horizontal momentum was corrected by 10°, as shown in Figure 15, and the vertical momentum was corrected by 7°, as shown in Figure 16. Consequently, the ratios of the radial, tangential, and axial momentums had changed as compared to SMG, taking the values: *m*_r_ = 0.474095, *m*_t_ = 0.789027, and *m*_a_ = 0.390731.

**Figure 15:**
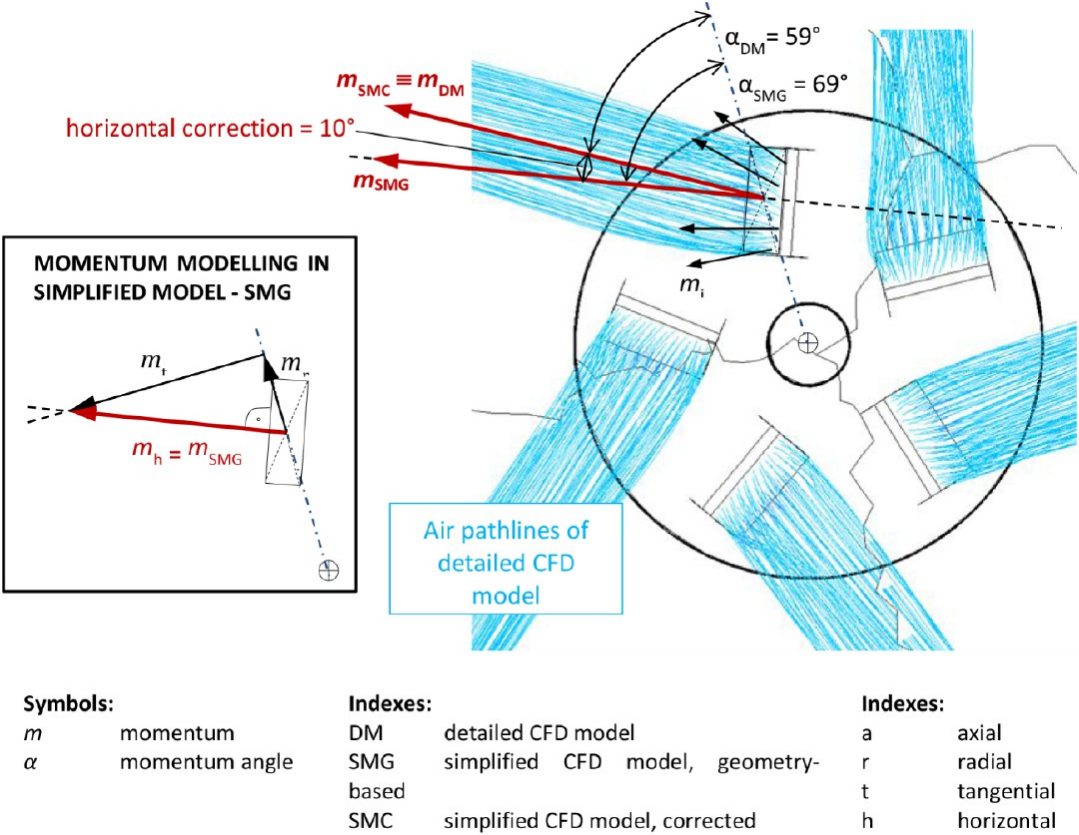
Comparison of horizontal jet between the detailed model (DM) and the geometry-based simplified model (SMG), and horizontal correction of the momentum angle in the corrected simplified model (SMC), using the SST *k-ω* turbulence model.

**Figure 16:**
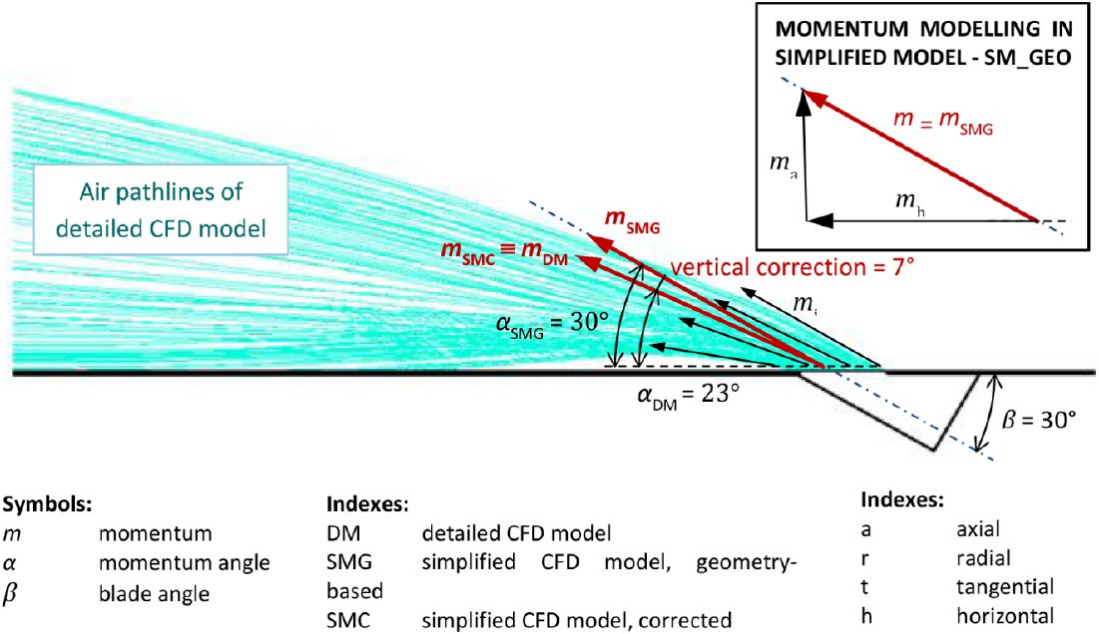
Comparison of vertical jet between the detailed model (DM) and the geometry-based simplified model (SMG), and vertical correction of the momentum angle in the corrected simplified model (SMC), using the SST *k-ω* turbulence model.

## 5 Indicators Used to Evaluate the CFD Models

Table 4 provides an overview of the indicators used to evaluate the CFD models. The matrix highlights which indicators were applied to compare PIV measurements with CFD results and which were used to compare the CFD models with one another. Quantitative indicators capture differences in parameters such as computation time, pathline angles, jet width, and boundary-layer thickness. Qualitative indicators were used to visually compare the shapes of turbulence intensity and air-velocity contours, complementing the information provided by the quantitative metrics.

**Table 4:**
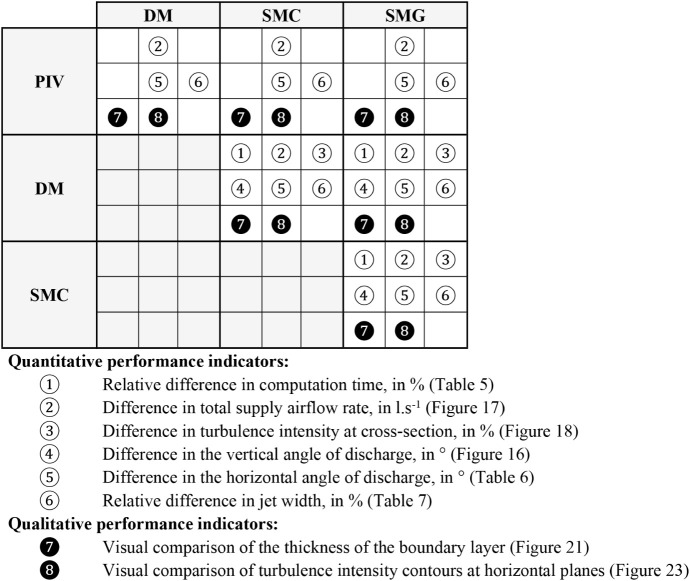
Overview of Indicators Used to Evaluate the CFD Models

## 6 Evaluation of the Accuracy of the CFD Models

Unless otherwise specified, the results for a horizontal plane refer to a representative plane located 20 mm above the diffuser face. Planes at 10 and 30 mm were also analyzed, but their trends were similar to those at 20 mm and are therefore not shown in detail.

All CFD simulations employed the SIMPLE pressure-velocity coupling algorithm, except Section 6 compares computation times for the SIMPLE and Coupled algorithms.

### 6.1 Relative Difference in Computation Time, in %

The longest computation time occurred for the DM combined with the RSM and the Coupled pressure-velocity coupling algorithm, which is defined as 100% in Table 5. Computation times for the remaining cases are expressed as percentages relative to this reference. The results indicate that the choice of pressure-velocity coupling algorithm affected computation time by several tens of percent, whereas the influence of the CFD model itself was only a few percent. The turbulence model exerted the strongest impact: using the RSM increased computation time by as much as a factor of 6 compared with the SST *k-ω* model.

**Table 5:** Computation Time for the 12 Cases Investigated

			Pressure-velocity coupling			
			Simple		Coupled	
			Turbulence model			
CFD model	Number of cells	Parameter	SST *k-ω*	RSM	SST *k-ω*	RSM
**Detailed (DM)**	274 919	No. of iterations	272	1 367	316	1 039
		Computation time	17%	64%	26%	100%
**Simplified (SMG)**	266 022	No. of iterations	321	1 503	399	1 071
		Computation time	12%	79%	31%	98%
**Simplified (SMC)**	266 022	No. of iterations	295	1 415	410	1 136
		Computation time	18%	68%	27%	96%

### 6.2 Difference in Total Air Supply Flow Rate

The total supply airflow rate determined from PIV at R125 was 7.16 l.s^–1^, approximately twice the primary flow measured in the air supply duct (3.11 l.s^–1^). This difference arises from the induction of secondary air (Figure 4).

CFD simulations were also used to determine the total flow rate at radii R85, R100, and R125. In all cases studied, the total air supply flow rate was predictably increasing with the radius (Figure 17). In every case, the PIV-based flow rate was lower than the CFD prediction, likely because PIV is two-dimensional and omits the axial (z) velocity component.

**Figure 17:**
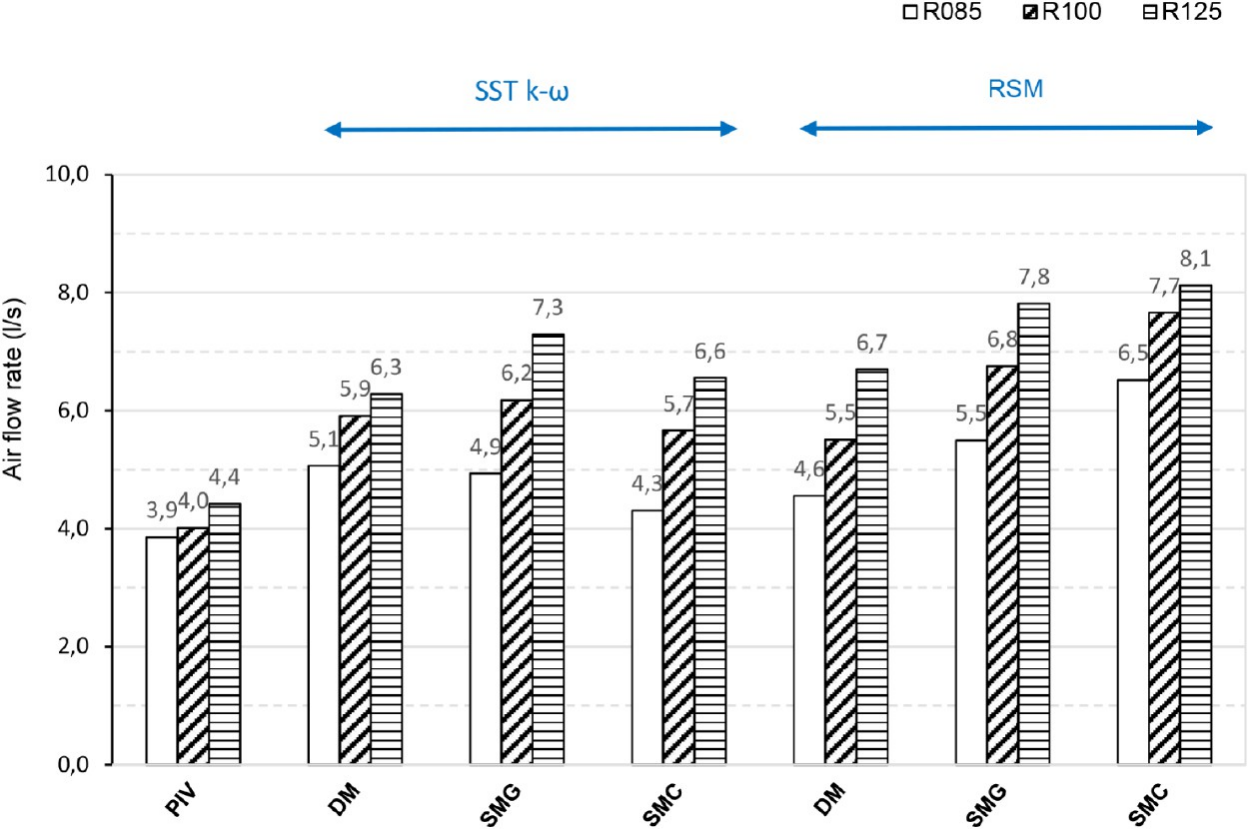
Total supply airflow rate obtained by PIV and CFD.

For the detailed CFD model (DM), the differences between the two turbulence models were minor. The geometry-based simplified model (SMG) produced results close to those of the DM at the smallest radius (R85) but showed a notable discrepancy at the largest radius (R125). The corrected simplified model performed relatively well with the SST *k-ω* turbulence model, yielding results close to the DM, whereas the RSM turbulence model substantially overestimated the airflow rate.

### 6.3 Relative Difference in Turbulence Intensity at the Vertical Cross-Section

The high density of the glycerin fog in the cross-section prevented the PIV system from detecting the turbulent structures needed to analyze the flow and generate vector fields. This is a general limitation of the PIV method. As a result, the turbulence intensity at the slot could not be measured directly, and the reliability of the investigated cases was assessed by comparing the simplified models with the detailed model.

Figure 18 presents the turbulence intensity calculated at cross-section A–A′. Error bars denote minimum and maximum values, while the columns indicate mean values. The minima and maxima of the simplified models fell approximately within the range of those of the detailed model, indicating that the assignment of turbulence characteristics at the supply openings in the simplified models was appropriate. A clear difference is evident between SMG and SMC, demonstrating that the angle corrections applied in SMC had a significant effect on turbulence intensity.

**Figure 18:**
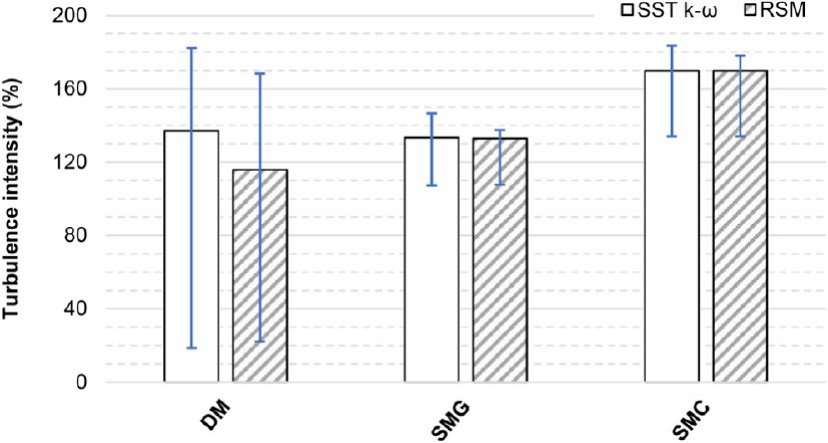
Turbulence intensity at cross sections A-Á obtained by CFD.

### 6.4 Difference in the Vertical Angle of Discharge, in °

No PIV measurements were available for the vertical discharge angle. Therefore, the reliability of the simplified models was assessed by comparison to the more precise DM. Figure 16 presents the results for the SST *k-ω* model. The vertical angle differed by 7° between SMG and DM, whereas no difference was observed between DM and SMC because SMC incorporated an angle correction based on the DM results. Within the individual CFD simulations, the choice of the turbulence model had a negligible effect on the vertical discharge angle.

### 6.5 Difference in the Horizontal Angle of Discharge, in °

The difference in the horizontal discharge angle was determined at the reference slot by superimposing the CFD-predicted air pathlines onto the PIV-measured air velocity vectors (Figure 19). Results for all investigated cases are summarized in Table 6. Both the detailed model (DM) and the simplified model SMC performed well, particularly when combined with the RSM turbulence model. In contrast, the simplified model SMG showed markedly lower accuracy with deviations of up to 14°.

**Figure 19:**
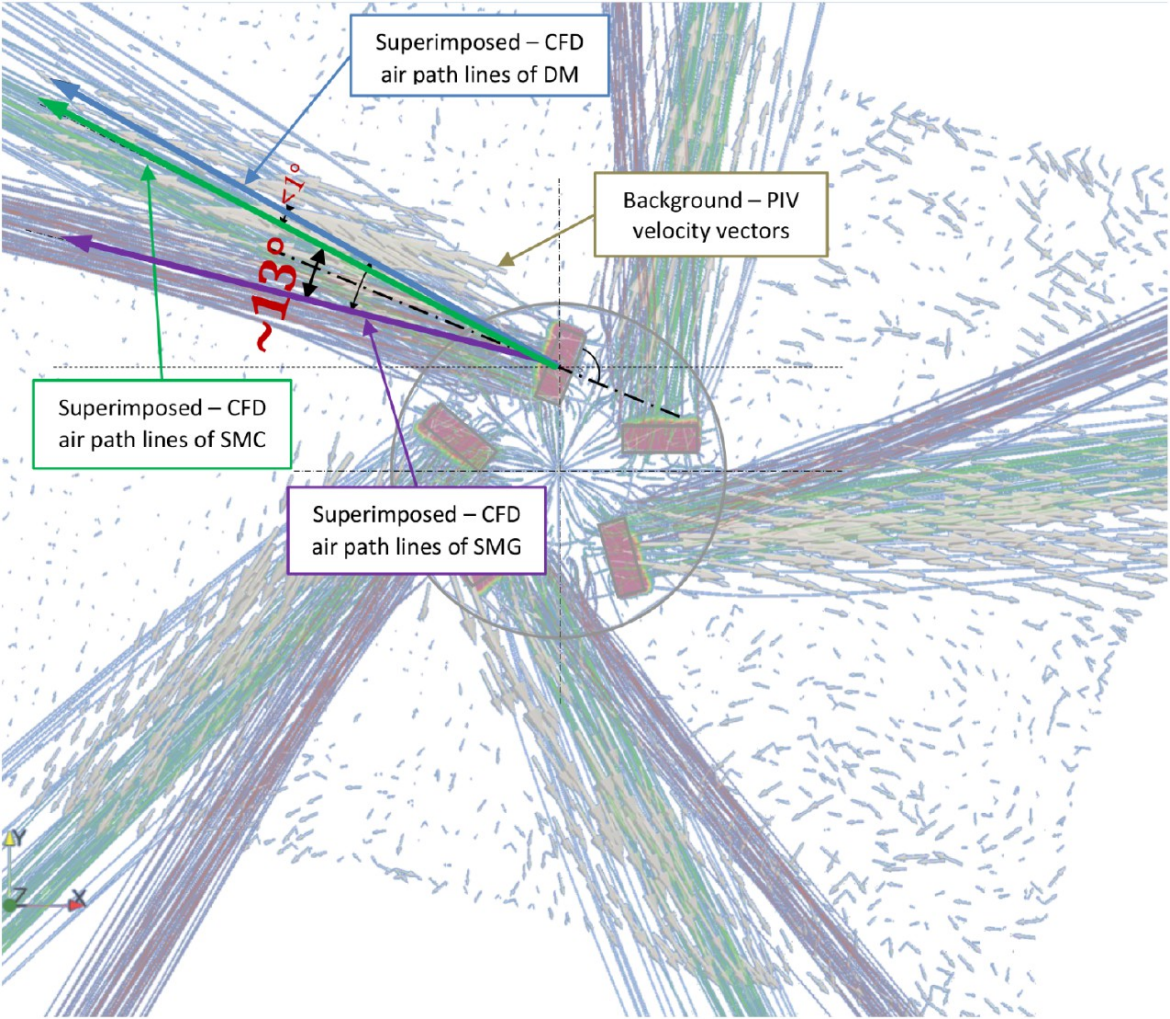
Determination of the difference in the horizontal angle of discharge (°) between PIV, the detailed model (DM), and the simplified models (SMG, SMC) using the RSM turbulence model.

**Table 6:** Difference in the Horizontal Angle of Discharge (°) Obtained by PIV and CFD

	Turbulence model	
CFD model/Case investigated	SST *k-ω*	RSM
**DM**	0.5	0.3
**SMG**	13.9	13.7
**SMC**	0.9	0.8

### 6.6 Relative Difference in Jet Width, in %

The relative difference in jet width was evaluated by comparing the air-velocity vector field obtained from PIV (Figure 20a) with the air pathlines predicted by CFD (Figure 20b and c). The velocity fields and pathlines in Figure 20 combine data from horizontal planes located 10, 20, and 30 mm above the diffuser face. As shown in Table 7, the differences among the three CFD models (DM, SMG, and SMC) were minor, whereas the discrepancies between the two turbulence models (RSM and SST *k-ω*) were substantial. These results indicate that the RSM turbulence model predicts the jet width more accurately than the SST *k-ω* model.

**Figure 20:**
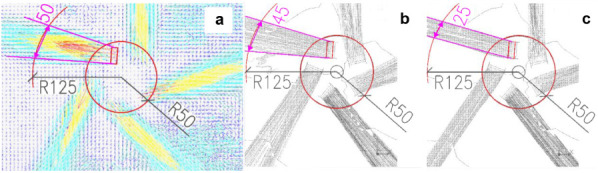
Determination of jet width (mm): a) PIV, b) DM_RSM, c) DM_SST *k-ω.*

**Table 7:** Relative Difference in Jet Width (%) Obtained by PIV and CFD

	**Turbulence model**	
CFD model/Case investigated	SST k-ω	RSM
**DM**	–50	–10
**SMG**	–50	10
**SMC**	–46	10

### 6.7 Relative Difference in Thickness of the Boundary Layer, in %

The air velocity boundary layer obtained from the PIV measurements is shown in Figure 21 as the solid red curve, which delineates the region where air velocity ≥ 0.2 m.s^–1^. After reaching its maximum thickness, the boundary layer gradually decreases as the airflow spreads and the flow diameter increases. The PIV boundary layer was derived by time-averaging the velocity field to smooth the inherent temporal fluctuations of the airflow.

**Figure 21:**
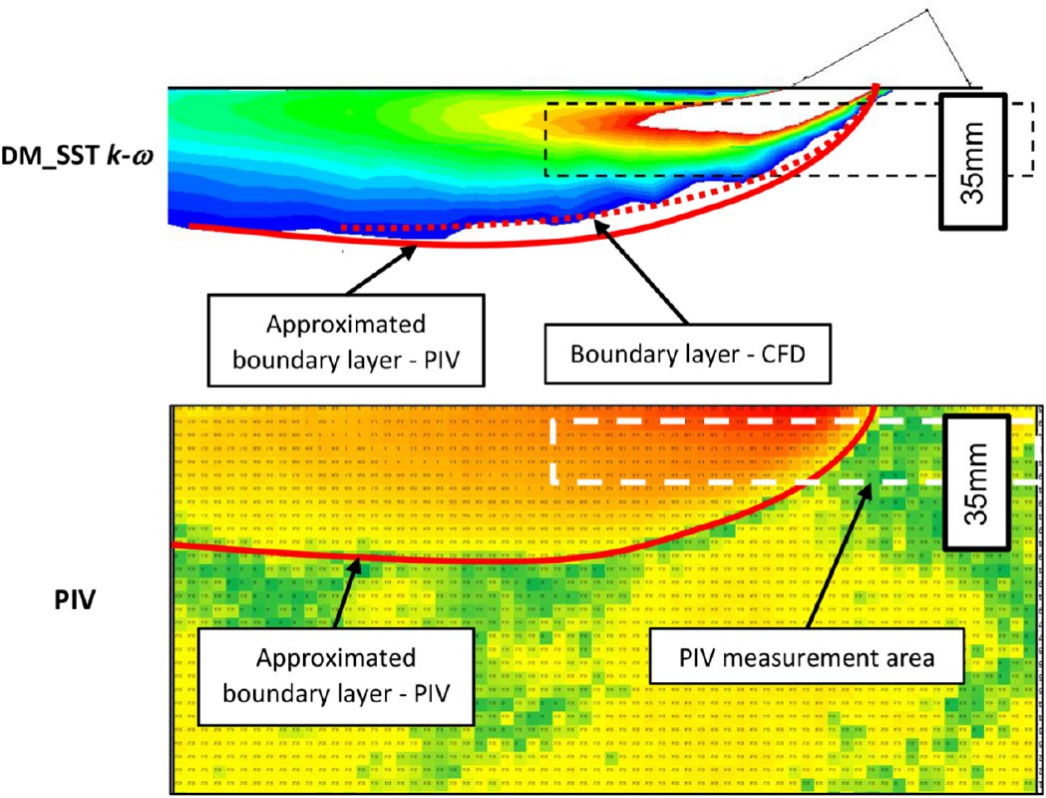
Air velocity boundary layer at cross-section A-Á obtained by PIV and DM.

The PIV-derived contour is compared with that obtained from CFD. In the experiment, the thickness of the boundary layer fluctuates over time, causing discrepancies in the boundary layer thickness between PIV and CFD. Generally, the boundary layer generated by CFD was in good agreement with the one obtained from the PIV measurement. CFD modeling succeeded to simulate the Coanda effect which describes jet attachment and modifies the near-wall flow and the apparent boundary-layer thickness.

### 6.8 Visual Comparison of Turbulence Intensity Contours at Horizontal Planes

The turbulence intensity contours obtained from PIV (Figure 22) are compared with the CFD results in Figure 23 at height z = 0.02 m. The simplified corrected model (SMC) exhibits significantly better agreement with both the detailed model (DM) and the PIV measurements than the simplified geometry-based model (SMG). The SST *k-ω* turbulence model shows better agreement with the PIV data than the RSM model.

**Figure 22:**
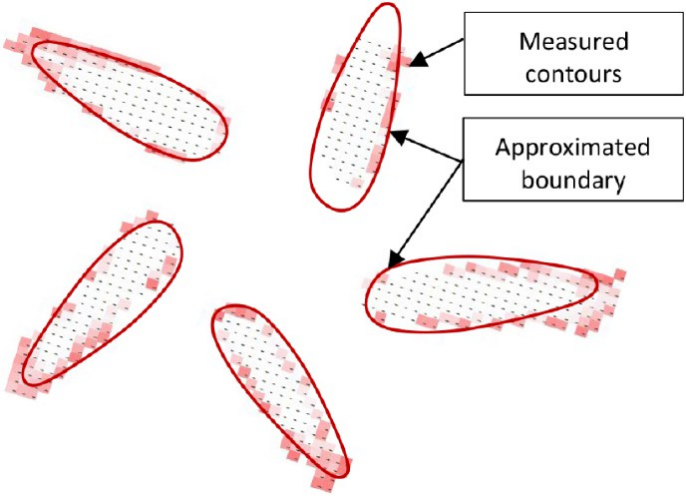
Turbulence intensity contours obtained by PIV (contour at 50% intensity).

**Figure 23:**
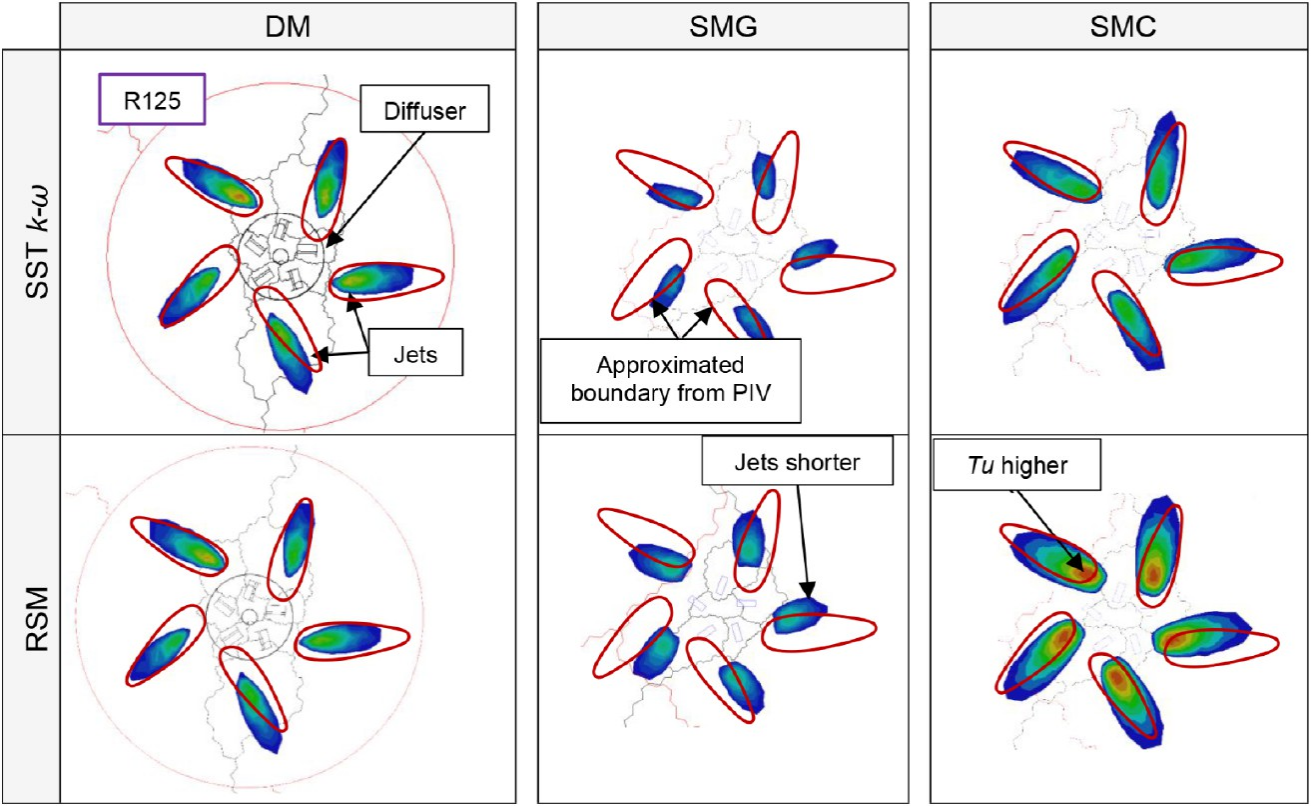
Visual comparison of turbulence intensity contours obtained by CFD and PIV (scale: 50% blue to 120% red).

## 7 Discussion

Performance evaluation of the investigated cases was based primarily on comparison with PIV measurements. For the detailed model (DM), the best results were obtained with the RSM turbulence model, which captured inhomogeneous turbulence more effectively than the SST *k-ω* model. the RSM better accounted for inhomogeneous turbulence. Overall, the comparison of PIV to the detailed CFD model has shown a reasonable match. Overall, the PIV–CFD comparison showed good agreement, so DM was used as the benchmark when measurements were unavailable, such as for turbulence intensity at the cross-section and for the vertical angle of discharge.

### 7.1 The Effect of Correction of Simplified CFD Model

The primary benefit of correcting the geometry-based simplified model (SMG) was the proper alignment of the horizontal and vertical discharge-momentum angles (see SMC in Figure 16 and Table 6). Moreover, SMC did not perform significantly worse than SMG for any of the evaluated performance indicators. Therefore, the corrected model (SMC) can be considered more reliable than the SMG.

### 7.2 The Effect of Turbulence Model

Two of the studies summarized in Table 1 examined the influence of different turbulence models on the CFD results. For a vortex diffuser, Aziz et al.^16^ tested the Standard *k-ε*, RNG *k-ε*, and Standard *k-ω* model, comparing their predictions with the temperature distribution measured in their own work and with the velocity distribution reported by Srebric and Chen.^7^ The Standard *k-ε* and Standard *k-ω* performed well, with the Standard *k-ω* model providing the best agreement, and were subsequently selected for further simulations. For a floor swirl diffuser, Yau et al.^19^ evaluated the Standard *k-ε*, Realizable *k-ε* and RNG *k-ε* turbulence models. Both the Standard *k-ε* and RNG *k-ε* models reproduced the centerline air-velocity pattern observed in measurements, but the Standard *k-ε* model achieved the closest match and was therefore adopted for further simulations. These findings help explain why the Standard *k-ε* model is the most frequently used in CFD studies of swirl diffusers. However, none of the studies in Table 1 compared a two-equation turbulence model with the elaborate six-equation RSM.

The alignment of the horizontal discharge angle with PIV measurements was slightly better for the RSM than for the SST *k-ω* model when combined with the corrected simplified model (SMC) (Table 6). The most notable advantage of RSM was its relatively small error in predicting the jet width, which agreed well with the PIV results. In contrast, SST *k-ω* showed reasonable performance in predicting boundary-layer thickness, turbulence intensity, and supply airflow rate, while requiring only a fraction of the computation time compared with RSM. The principal limitation of SST *k-ω*, however, was its tendency to predict an overly narrow flow jet (Table 7). This shortcoming can be attributed to its neglect of turbulence anisotropy, specifically, the damping of turbulence fluctuations perpendicular to the ceiling, as noted by Schälin and Nielsen.^33^ This effect may be even more pronounced at room scale.

### 7.3 The Effect of Pressure-Velocity Coupling Algorithm

Most of the studies listed in Table 1 do not specify the algorithm used for pressure-velocity coupling, and none compare the performances of the SIMPLE and Coupled schemes. In the present study, these two algorithms were evaluated with respect to computational cost. The Coupled scheme required more iterations and was approximately 9% more time-consuming for the SST *k-ω* turbulence model and 19% more time-consuming for the RSM turbulence model.

## 8 Conclusions

The present study reports experience in developing simplified CFD models of swirl diffusers with angled slots arranged in a ring, using momentum modeling at the air supply. It also demonstrates the applicability of 2D PIV for evaluating the CFD model performance. All CFD models were tested with both the high-fidelity RSM and the less computationally demanding SST *k-ω* turbulence models. In addition, the SIMPLE and Coupled pressure-velocity coupling algorithms were compared in terms of computational cost. The main conclusions are summarized as follows:

- To create the corrected simplified model (SMC), a geometry-based simplified model (SMG) was first developed with supply opening dimensions identical to those of the actual diffuser slots. The model’s radial, axial, and tangential momentum components were then adjusted to enhance reliability. For the investigated swirl diffuser with a slot angle of 30°, the momentum angle was corrected by 10° horizontally and 7° vertically to achieve close agreement with the detailed CFD model, which had been validated against PIV measurements. Adjusting the momentum angles improved the reliability of the simplified model.
- Among the simplified models tested, the corrected model (SMC) produced results comparable to those of the detailed CFD model (DM) combined with the RSM turbulence model, while substantially reducing modeling and computation time. SMC therefore offers a favorable balance between reliability and computational efficiency, making it suitable for practical applications.
- The 2D PIV measurements supported evaluation of both the detailed and simplified CFD models, enabling direct comparison of measured and simulated turbulence intensity values, air-velocity boundary layers, jet widths, and horizontal discharge angles.
- The main limitations of the PIV measurements were the uneven fog distribution across the measurement plane and the restriction of 2D PIV to simultaneous measurement of only two of the three flow components. These factors introduced uncertainties in predicting the supply airflow rate.
- Future work should include recommendations for correcting momentum angles across different discharge angles, supply airflow rates, and diffuser sizes and types, to facilitate the practical application of CFD in modeling swirl diffusers. Subsequent studies could also extend these findings to the whole-room scale.

## ⊥

Veveří 331/95, 60200, Brno, Czech Republic
